# Associations of reverse triiodothyronine serum levels with anxiety, depression, and health related quality of life after experiencing acute ischemic stroke

**DOI:** 10.3389/fneur.2024.1474490

**Published:** 2025-01-07

**Authors:** Saulius Taroza, Helene Plamondon, Aurelija Podlipskyte, Nijole Kazukauskiene, Nicolas Francisco Narvaez Linares, Marilou Poitras, Julius Burkauskas, Narseta Mickuviene

**Affiliations:** ^1^Laboratory of Behavioral Medicine, Neuroscience Institute, Lithuanian University of Health Sciences, Palanga, Lithuania; ^2^Behavioural Neuroscience Group, School of Psychology, University of Ottawa, Ottawa, ON, Canada

**Keywords:** reverse T3, stroke, depression, anxiety, health-related quality of life

## Abstract

**Aim:**

This study intended to explore associations of reverse triiodothyronine (rT3) with emotional disturbances and health-related quality of life (HRQoL) after experiencing acute ischemic stroke (AIS).

**Materials and methods:**

Serum samples from individuals with AIS were collected on admission to three Lithuania stroke centers and investigated for free tetraiodothyronine, free triiodothyronine (fT3), rT3, and thyroid stimulating hormone levels. At discharge, emotional disturbance was evaluated using the Hospital Depression and Anxiety Scale (HADS), and HRQoL using the EQ-5D-5L scale.

**Results:**

Analyses included 159 individuals (59.7% male and 40.3% female; mean (SD) aged 66.4 [10.3] years), 52.83% of which showed increased rT3 levels upon admission. After adjustment for age, sex, National Institutes of Health Stroke Scale scores, previous stroke, modified Rankin Scale before AIS ≤ 2, and diabetes mellitus, multivariable linear regression revealed negative associations of rT3 with HADS total score (*β* = −0.163; *p* = 0.046) and HADS-D subscale score (*β* = −0.187; *p* = 0.019). Analyses supported a positive relationship between the fT3 ratio to rT3 with HADS-D score (*β* = 0.157; *p* = 0.046) and rT3, rT3 × fT3 product with EQ-5D index score (*β* = 0.157; *p* = 0.044 and *β* = 0.179; *p* = 0.023, respectively).

**Conclusion:**

We found that individuals who experienced AIS and had higher levels of rT3 at hospital admission had less emotional disturbance and better HRQoL when discharged.

## 1 Introduction

While stroke is the third leading cause of disability worldwide, it is the leading cause of neuropsychiatric conditions, such as depression (1). In addition, involving changes in regulatory brain processes, stroke has been associated with reductions in self-reported health-related quality of life (HRQoL) (2).

Through associated dysregulation of neuroendocrine responses, stroke represents a potent physiological stressor known to affect thyroid function (3). For instance, in a third of acute stroke patients, serum 3,3′,5-triiodothyronine (T3) levels were detected below the norm (4). This disease-non-specific thyroid response or non-thyroidal illness syndrome (NTIS) in a broader sense manifests through lowered T3 levels (or low-T3 syndrome), elevated 3,3′,5′-triiodothyronine (reverse T3 or rT3) and normal or lowered thyroid stimulating hormone (TSH) (5). However, recent research supports the response of the thyroid system to stroke to be more complex. Reduced free T3 (fT3) and / or low total T3 in the acute period after a stroke are associated with more severe neurological impairments, poorer global stroke outcomes, increased mortality (4), and post-stroke depression (6). Furthermore, evidence supports an association between increased free tetraiodothyronine (fT4) and poorer acute ischemic stroke (AIS) outcomes (7). Other researchers have found inconsistencies in TSH to affect stroke outcomes (6, 8, 9).

Notably, there are numerous studies have evaluated the association of serum rT3 levels with the severity of illness and its prognosis. For instance, while a study revealed increased rT3 to positively correlate with mortality in the intensive care unit (10), another supported an independent role of rT3 in predicting mortality in advanced-age individuals (11). The measured total T3 to rT3 ratio, reflecting tissue hormone turnover, was also associated with the prognostic of survival from the first day of critical illness (12). A subsequent study similarly uncovered a negative correlation between the fT3 to rT3 ratio and the heart failure (13). Interestingly, recent research on individuals with COVID-19 found higher fT3 × rT3 product value to be related to higher mortality rates, while an inverse relationship was found between elevated rT3 and increased mortality and disease severity (14). To our knowledge, this study was the first one to look for the value of fT3 × rT3 product as a disease severity marker. Moreover, increased rT3 was found in acute depression with severe symptoms (15).

Looking at the rT3 serum levels associations with stroke type and brain injury there is older paper in Chinese mentioning that rT3 abnormalities were more commonly found in individuals with hemorrhagic stroke versus ischemic and positive rT3 association with brain lesion volume (16). Another article in Polish mentioned higher rT3 as a possible marker of mortality after AIS (17).

It is known, that rT3 as a metabolite of T4, the main thyroid pro-hormone, associates with nuclear thyroid hormone (TH) receptors a few hundred times weaker than the main active TH – T3, therefore its action through this mechanism in normal conditions is negligible (18). But there are some clues on rT3 action through extra-nuclear receptors in certain proliferative conditions (19). In addition to this, new proposition suggests a possible non-genomic rT3 action during NTIS in COVID-19 infection, when there are elevated levels of this substance, with pathological effect on vascular and coagulation system (20). Information regarding its potential activity in the brain is restricted with experimental studies involving rT3 administration at supra-physiological concentrations. A previously performed study on thyroidectomized rats showed, that intravenously injected rT3 could be associated with regulation of iodothyronine deiodinase type II activity suppressing it within the cortex (21). Exposure of rats’ hippocampus with rT3 restored chemical dysregulation because of experimentally induced hypothyroidism (22). Another experimental study on Sprague–Dawley rats with induced brain ischemia through middle cerebral artery occlusion established, that additionally intravenously applicated rT3 had potential role in attenuating cerebral ischemia and reperfusion injury through suppression of oxidative stress (23). Mentioned experimental studies provide some clues as to the possible activity of rT3, at least in certain conditions.

Overt thyroid disorder has huge negative impact on individuals’ psychology and quality of life (24, 25). Further on, it’s important to underline that optimal TH and TSH levels are not equal to their reference values (26). For example, there are data linking high-normal TSH with severity of major depression (27). One of possible explanations in relation to TSH could be its incapabilty to fully reflect thyroid status in different organs (28). Probably different conditions have their own optimal ranges of thyroid profile (26, 28).

These findings illustrate the complex nature of the thyroid axis reaction, peripheral thyroid hormone metabolism changes to stroke, and unresolved issues regarding the value of rT3 in stroke prognosis: are there significant associations between rT3 and non-fatal stroke outcomes, and, if so, are higher rT3 levels associated with worse stroke outcomes? To further investigate these questions, this study aimed to closely evaluate the associations between serum rT3 levels and its ratio to fT3, and the product of these variables (fT3 × rT3) with emotional status and HRQoL in the acute period after an AIS without overt thyroid disorder.

## 2 Materials and methods

### 2.1 Study procedure

This study is a follow-up of prior work investigating thyroid axis hormone levels correlations with AIS outcomes (29). Individuals with experienced AIS (*N* = 612) according to the World Health Organization criteria (30), admitted to the three Lithuanian stroke centers (i.e., Klaipėda University Hospital, Hospital of the Lithuanian University of Health Sciences Kauno Klinikos, and Klaipėda Seamen’s Hospital) during the years 2013–14 and 2016 were invited to participate in the study. The study protocol allowed the inclusion of all individuals between eighteen and eighty years of age who presented to the hospital within the first 2 days of symptom onset. The diagnosis of AIS was confirmed by brain-computer tomography or magnetic resonance imaging.

Individuals were excluded from the study due to: (a) anamnesis of thyroidopathy and/or current use of thyroid affecting substances (i.e., amiodarone, heparin, iodinated contrast, carbamazepine, thyroxin, thyrostatic; *n* = 99); (b) malignancy, severe infection, renal or hepatic disease, severe exhaustion, including premorbid swallowing disorder and malnutrition (*n* = 58); (c) thyroid blood test results outside the defined boundaries as listed below (*n* = 23); (d) refusal to participate (*n* = 130); (e) admission >2 days after the onset of AIS (*n* = 60); (f) death during follow-up (*n* = 12); (g) impairment of communication (*n* = 45); and (h) did not perform the assessment of emotional disturbance and complete the HRQoL (*n* = 26). Only 159 individuals could be included.

The study was conducted in accordance with the Declaration of Helsinki and the requirements of the Regional Biomedical Research Ethics Committee (licenses: P1-BE-2-11/2013 and P2-BE-2-11/2013). Individuals were included only after giving written consent for participation in this study.

### 2.2 Study design

The following data was collected on admission: (a) age; (b) sex; (c) severity of neurological deficit according to the National Institutes of Health Stroke Scale (NIHSS); (d) pre-stroke dependency based on modified Rankin Scale (mRS); (e) antithrombotic use; and (f) vascular risk factors, such as arterial hypertension (AH), atrial flutter (AF), active smoking, diabetes mellitus (DM), previous stroke, previous myocardial infarction (MI).

Blood samples for thyroid profile were taken on admission within the first 48H of symptom onset. Individuals’ emotional status and HRQoL were assessed at the end of the inpatient period – usually between the seventh and tenth day.

### 2.3 Assessment of thyroid axis hormones

Serum levels of TSH, fT3, rT3, and fT4 were measured. The serum was separated from the blood by centrifugation at 3000 g and frozen at −70 oC. Concurrent evaluation of TSH, fT3 and fT4 was performed with an electrochemiluminescence immunoassay analyser (Advia Centaur XP 2016; Siemens Osakeyhtiö, Espoo, Finland) and rT3 with an enzyme immunoassay (ELISA) rT3 kit (EQ 1016-9601-9, EUROIMMUN, Germany). Participants included in analyses had hormones in the following ranges: TSH 0.1–10 mIU/L (involved within normal – 0.55–4.78 mIU/L – limits and with clinically insignificant deviations); fT3 ≤ 4.23 pg./mL (involved within normal – 2.02–4.23 pg./mL – limits and lower – for low-T3 syndrome cases); rT3 involved with any value, which in our case ranged from 0.11 to 0.95 ng/mL; and fT4 0.75–1.48 ng/dL (involved cases only within normal limits).

### 2.4 Evaluation of anxiety/depression and HRQoL

Emotional disorder was evaluated using the validated Lithuanian version of the Hospital Anxiety and Depression Scale (HADS) questionnaire (31). This scale is designed to assess symptoms of depression (HADS-D subscale) and anxiety (HADS-A subscale).

HRQoL was assessed using the EQ-5D-5L instrument, which was recently validated in the Lithuanian stroke population (32). The descriptive part of EQ-5D-5L instrument evaluates health in 5 dimensions with a possible choice of 5 levels. A summary index score can be assigned to each health state. The EQ-5D index was chosen to represent HRQoL.

Both questionnaires have been proven culturally sensitive and have already been used in previous studies in Lithuanian adult populations (32, 33).

### 2.5 Statistical analysis

Statistical analyses were performed using the IBM SPSS Version 29.0.0.0 (Chicago, IL, United States). The normally distributed data were expressed as mean ± standard deviation (SD). Other data were expressed as medians with interquartile ranges indicated in parentheses. Univariate and multivariable linear regression was performed to evaluate associations of rT3, fT3/rT3, and fT3 × rT3 with emotional disturbance and HRQoL. Given the exploratory nature of our work, we applied Bonferroni correction for multiple comparisons in the correlational analyses part (3 predictors in 4 independent models, p.05 /3 = 0.017), which served as a preliminary “screening” phase to identify potential relationships for further investigation using regression models.

Multivariable linear regression analysis was adjusted for six available confounders evaluated during admission (age, sex, NIHSS score, previous stroke, mRS before AIS ≤ 2, and DM). A minimum number of observations per variable of at least twenty-five was chosen to detect reasonable size effect. Statistical tests were two-tailed, and the significance level was *p* < 0.05.

## 3 Results

The main characteristics of all 159 included individuals and subdivisions into two groups according to normal or elevated rT3, are presented in Table 1. We found that slightly more than half (52.83%) of included individuals with experienced AIS had increased rT3 on admission. Individuals with rT3 elevated had less severe neurologic deficit according to NIHSS (*p* = 0.020) and less commonly experienced previous stroke (*p* = 0.029). A minority of individuals (*n* = 7) presented with low-T3 syndrome. Fourteen individuals had lowered TSH. fT3 to rT3 ratio was higher in rT3 normal group. HADS-D score was lower (*p* = 0.015), but EQ-5D index score was higher (*p* = 0.038) in rT3 elevated group.

**Table 1:** Main characteristics of all included individuals according to rT3 serum levels.

Sample size	All	rT3 normal (between 0.08 and 0.31 ng/mL)	rT3 elevated (> 0.31 ng/mL)	*p*
	159	75	84	
Demographic factors				
Age, years, mean ± SD	66.4 ± 10.3	66.6 ± 10.3	66.2 ± 9.8	0.795
Sex, Female, *n* (%)	64 (40.3)	32 (42.7)	32 (38.1)	0.557
Sex, Male, *n* (%)	95 (59.7)	43 (57.3)	52 (61.9)	
NIHSS score, median (IQR)	6.0 (3.0–11.0)	7.0 (4.0–12.0)	5.0 (3.0–8.75)	**0.020**
mRS before AIS ≤ 2, *n* (%)	151 (95.0)	70 (93.3)	81 (96.4)	0.373
Used antiaggregant, *n* (%)	46 (30.1)	22 (31.4)	24 (28.9)	0.736
Used anticoagulants, *n* (%)	19 (13.9)	8 (13.6)	11 (14.1)	0.927
Vascular risk factors				
AH, *n* (%)	108 (71.5)	49 (72.1)	59 (71.1)	0.895
AF, *n* (%)	48 (32.0)	23 (33.3)	25 (30.9)	0.747
Smoking, *n* (%)	32 (20.5)	11 (15.1)	21 (25.3)	0.114
DM, *n* (%)	23 (14.7)	11 (14.9)	12 (14.6)	0.968
Previous stroke, *n* (%)	29 (18.2)	19 (25.3)	10 (11.9)	**0.029**
Previous MI, *n* (%)	15 (9.6)	4 (5.4)	11 (13.4)	0.090
Thyroid test results on admission				
TSH (mIU/L), median (IQR)	1.22 (0.75–2.05)	1.24 (0.75–2.07)	1.21 (0.75–2.01)	0.903
fT3 (pg/mL), mean ± SD	2.80 ± 0.48	2.73 ± 0.42	2.86 ± 0.56	0.080
rT3 (ng/mL), mean ± SD	0.34 ± 0.13	0.23 ± 0.05	0.43 ± 0.11	**<0.001**
fT4 (ng/dL), mean ± SD	1.26 ± 0.19	1.25 ± 0.18	1.27 ± 0.04	0.519
Low fT3, *n* (%)	7 (4.4)	5 (6.7)	2 (2.4)	0.189
Low TSH, *n* (%)	14 (8.8)	9 (12.0)	5 (5.9)	0.061
fT3/rT3 ratio, mean ± SD	9.55 ± 4.03	12.5 ± 3.84	6.88 ± 1.56	**<0.001**
fT3 × rT3, mean ± SD	0.96 ± 0.52	0.63 ± 0.17	1.25 ± 0.55	**<0.001**
HADS and EQ-5D index estimate on discharge				
HAD score, median (IQR)	9.0 (6.0–14.0)	9.5 (6.0–15.3)	8.0 (6.0–12.0)	0.091
HADS-A score, median (IQR)	4.0 (2.0–7.0)	4.0 (2.0–7.0)	4.0 (2.0–6.0)	0.840
HADS-D score, median (IQR)	4.0 (2.0–8.0)	5.0 (3.0–10.0)	4.0 (2.0–6.0)	**0.015**
EQ-5D index, median (IQR)	0.81 (0.47–0.93)	0.72 (0.28–0.92)	0.83 (0.65–0.93)	**0.038**

AF, atrial fibrillation; AH, arterial hypertension; AIS, acute ischemic stroke; DM, diabetes mellitus; fT3, free triiodothyronine; fT4, free tetraiodothyronine; HADS, Hospital Anxiety and Depression Scale; HADS-A, Hospital Anxiety and Depression Scale-Anxiety subscale; HADS-D Hospital Anxiety and Depression Scale-Depression subscale; IQR, interquartile range; MI, myocardial infarction; mRS, modified Rankin Scale; NIHSS, National Institutes of Health Stroke Scale; SD standard deviation; rT3, reverse triiodothyronine; TSH, thyroid stimulating hormone. Numbers in bold indicate significant differences, *p* < 0.05.

The results of standard linear univariate and multivariable regression analyses are shown in Table 2. Univariate regression revealed negative associations between rT3, fT3 and rT3 product HADS-D was in reverse association with rT3 (*β* = −0.228; *p* = 0.004). Regarding EQ-5D index it was in positive relation with rT3 (*β* = 0.190; *p* = 0.016) and fT3 × rT3 (*β* = 0.225; *p* = 0.004). Multivariable analysis showed negative associations of rT3 with HADS total and HADS-D subscale scores (*β* = −0.163; *p* = 0.046) – elevated rT3 at admission was associated with reduced emotional disturbance on discharge. Notably, a positive association was established between the fT3 to rT3 ratio and the HADS-D subscale score (*β* = 0.157; *p* = 0.046). Regarding HRQoL, we uncovered positive associations between EQ-5D index score established upon discharge and rT3 (*β* = 0.157; *p* = 0.044) and fT3 × rT3 product (*β* = 0.179; *p* = 0.023, respectively) on admission.

**Table 2:** Univariate and multivariable linear regression of the contribution of thyroid profile on admission in AIS patients to emotional status and EQ-5D index at discharge.

Variables	Univariate linear regression				Multivariable linear regression				
	B	*β*	[95 %CI B]	*p*	*R* ^2^	B	*β*	[95 %CI B]	*p*
HADS^a^									
rT3	−9.03	−0.187	[−16.57 to −1.49]	0.019	0.080	−7.85	−0.163	[−15.56 to −0.140]	**0.046**
fT3/rT3	0.209	0.134	[−0.037 to0.456]	0.095	0.073	0.215	0.138	[−0.034 to 0.980]	0.089
fT3 × rT3	−1.97	−0.162	[−3.88 to −0.062]	0.043	0.070	−1.57	−0.130	[−3.55 to 0.406]	0.118
HADS–A^a^									
rT3	−2.08	−0.072	[−6.65 to 2.50]	0.371	0.027	−2.20	−0.077	[−6.94 to 2.53]	0.359
fT3/rT3	0.061	0.065	[−0.088 to 0.209]	0.420	0.026	0.060	0.065	[−0.092 to 0.213]	0.433
fT3 × rT3	−0.518	−0.071	[−1.67 to 0.634]	0.376	0.028	−0.581	−0.080	[−1.79 to 0.629]	0.344
HADS–D^a^									
rT3	−6.95	−0.228	[−11.66 to −2.24]	**0.004**	0.141	−5.65	−0.187	[−10.33 to −0.958]	**0.019**
fT3/rT3	0.149	0.151	[−0.006 to 0.304]	0.059	0.132	0.154	0.157	[0.003–0.306]	**0.046**
fT3 × rT3	−1.45	−0.189	[−2.65 to −0.255]	0.018	0.124	−0.993	−0.131	[−2.20 to 0.217]	0.107
EQ–5D index^a^									
rT3	0.480	0.190	[0.089–0.870]	**0.016**	0.150	0.394	0.157	[0.010–0.779]	**0.044**
fT3/rT3	−0.003	−0.039	[−0.160 to 0.010]	0.627	0.128	−0.004	−0.050	[−0.017 to 0.008]	0.517
fT3 × rT3	0.143	0.225	[0.045 to 0.240]	**0.004**	0.156	0.113	0.179	[0.016–0.211]	**0.023**

AIS, acute ischemic stroke; fT3, free triiodothyronine; HADS, Hospital Anxiety and Depression Scale; HADS-A, Hospital Anxiety and Depression Scale-Anxiety subscale; HADS-D Hospital Anxiety and Depression Scale-Depression subscale; mRS, modified Rankin Scale; NIHSS, National Institutes of Health Stroke Scale; rT3, reverse triiodothyronine. Statistical analysis: I recommend using corrections for multiple comparisons in regression analyses. We Bonferroni correction for multiple comparisons in univariate regression analyses (*p*=0.017).

a Adjusted for age, sex, NIHSS score, previous stroke, mRS before AIS ≤ 2, and DM. Numbers in bold indicate significant differences, *p* < 0.05.

## 4 Discussion

The relationship between elevated rT3 and reduced emotional disturbance and improved HRQoL is intriguing and contra-intuitive as it contrasts the classical NTIS statement where elevated rT3 in the critical acute period predicted worse outcomes (5). We were unable to find any study linking rT3 with HRQoL or emotional disturbance after stroke, so we have nothing to directly compare our results with. One older publication of rT3 in affective disorders with included 32 individuals suffering from major depression established elevated rT3 in those with the most pronounced symptoms but no difference was found between depression affected and euthymic with previously experienced affective condition or healthy individuals (15). Here, the discrepancy with our study results may be due to the small number of individuals included and the psychiatric condition of inorganic origin.

Based on more recent and available published studies linking rT3 to disease outcomes it should be emphasized that previous studies examining associations of rT3 usually used mortality as an outcome (10–12). Therefore, our findings could be related to the inclusion of surviving individuals, which brings a different perspective to data interpretation. In this context, a more complex profile of thyroid system responses to illness—in relation to rT3—is supported by a recently published study that included COVID-19 infected individuals (14). The latter study revealed higher rT3 serum levels on admission in survived versus non-survived and more often increase in rT3 serum levels in non-critical versus critical individuals. These findings coincide with ours – higher rT3 was more frequent in individuals with lower neurological deficit versus with higher neurological deficit on admission. Enigmatic relationship between higher rT3 serum levels and better outcome presumably could be explained by activity of TH metabolizing proteins – normal iodothyronine type 1 deiodinase activity and / or failure of iodothyronine type 3 deiodinase activation (14). It seems that there is still long way to go before we have a clearer understanding of how much and how this hormone affects / reflects stroke physiology and outcomes. From our research we can only speculate, that increased rT3 serum levels could be in some ways associated with its protective role on damaged brain.

Our work is innovative in discovering a new association between elevated levels of rT3 and lower emotional disturbances, as well as enhanced HRQoL, upon discharge in patients with AIS. This emphasises the intricate nature of thyroid axis reactions and peripheral rT3, and T3 turnover changes after stroke. The results emphasise the need for more study to enhance the predictive significance of rT3 and clarification of direction of possible associations with different stroke outcomes, including comparison of AIS survivals with non-survivals and / or transient ischemic attack experienced individuals, which might result in more effective control of thyroid gland reactions and the development of new techniques for preventing stroke in the future. Gaining insight into the correlation between rT3 and mental health after a stroke may greatly alleviate the challenges of rehabilitation, highlighting the possibility of preventive measures to improve patient outcomes and overall well-being.

## 5 Strengths and limitations

The study is strengthened by three different stroke centers from the same country. Some limitations of the study include the limited number of tested individuals, blood collection at different daily intervals, and the omission of other important factors for the measured outcomes, including brain lesion topography and volume and premorbid wellness assessments.

## 6 Conclusion

Our research established associations of higher serum rT3 with reduced emotional disturbances and better HRQoL on discharge, contributing to other research supporting complex thyroid axis responses following ischemic stroke. These findings warrant further investigations in this field, particularly to refine the value of rT3 in stroke prognosis.

## Data Availability

The raw data supporting the conclusions of this article will be made available by the authors, without undue reservation.
